# Multicenter randomized controlled trial of intensive uric acid lowering therapy for CKD patients with hyperuricemia: TARGET-UA

**DOI:** 10.1007/s10157-024-02483-w

**Published:** 2024-03-26

**Authors:** Tetsuya Yamamoto, Masato Kasahara, Kenji Ueshima, Shiro Uemura, Naoki Kashihara, Kenjiro Kimura, Tsuneo Konta, Tetsuo Shoji, Akira Mima, Masashi Mukoyama, Yoshihiko Saito

**Affiliations:** 1 Gyoumeikan Hospital, Osaka, Japan; 2 Nara Medical University, Kashihara, Japan; 3 Ujitakeda Hospital, Uji, Japan; 4 Kawasaki Medical School, Okayama, Japan; 5 JCHO Tokyo Takanawa Hospital, Tokyo, Japan; 6 Yamagata University, Yamagata, Japan; 7 Osaka Metropolitan University, Osaka, Japan; 8 Osaka Medical and Pharmaceutical University, Osaka, Japan; 9 Kumamoto University, Kumamoto, Japan

**Keywords:** Chronic kidney disease (CKD), Uric acid-lowering therapy, Renal function, Urine albumin to the creatinine ratio, Topiroxostat

## Abstract

**Background:**

We investigate whether Intensive uric acid (UA)-lowering therapy (ULT) provides increased renal protection compared with standard therapy in chronic kidney disease (CKD) patients.

**Methods:**

This was a multicenter randomized controlled trial. Only CKD patients with hyperuricemia were included in this study. The participants were randomly assigned to either the Intensive therapy group (target serum UA level ≥ 4.0 mg/dL and < 5.0 mg/dL) or the standard therapy group (serum UA level ≥ 6.0 mg/dL and < 7.0 mg/dL). ULT was performed using topiroxostat, a non-purine-type selective xanthine oxidase inhibitor. The primary endpoint was change in the logarithmic value of urine albumin to the creatinine ratio (ACR) between baseline and week 52 of the treatment.

**Results:**

Three hundred fifty-two patients were included in the full analysis set. In the Standard therapy group, mean serum UA was 8.23 mg/dL at baseline and 6.13 mg/dL at 52 weeks. In the Intensive therapy group, mean serum UA was 8.15 mg/dL at baseline and 5.25 mg/dL at 52 weeks. There was no significant difference in changes in log ACR at 52 weeks between the Intensive therapy and the Standard therapy groups.

**Conclusion:**

This study did not reveal the benefit of Intensive ULT to improve albuminuria levels.

(UMIN000026741 and jRCTs051180146).

**Supplementary Information:**

The online version contains supplementary material available at 10.1007/s10157-024-02483-w.

## Introduction

In many countries, prevention of chronic kidney disease (CKD) is an important issue. Hyperuricemia is known to reduce renal function through endothelial dysfunction [1], renal tubular injury [2], and glomerular sclerosis [3]. Clinical studies demonstrated that lowering of serum uric acid (UA) is associated with renal protection [4–9], lower blood pressure [6, 7], improved endothelial function [10], and a reduction in the risk of cardiovascular events [11]. A cohort study in Japan demonstrated that odds ratios [95% CI] for developing CKD with UA ≤ 4.0 mg/dL as a reference were 1.21 [0.84, 1.74] for UA 4.1–4.9 mg/dL, 1.47 [1.01, 2.17] for UA 5.0–5.8 mg/dL, and 2.10 [1.37, 3.23] for UA ≥ 5.9 mg/dL [12]. In addition, Goicoechea et al. reported that allopurinol, a xanthine oxidase inhibitor (XOI), lowered serum UA and reduced the decline in estimated glomerular filtration rate (eGFR) [11]. Based on this evidence, the guidelines of the Japanese Society of Nephrology recommend that UA level should be controlled below 6.0 mg/dL [13]. However, the optimal target level of serum UA for renal protection has not been determined. Observational studies showed that a UA level of 5.0 mg/dL or less was more protective against the progression of renal dysfunction compared with a level of 6.0 mg/dL or more [14, 15]. This data suggest that a UA target level less than 5.0 mg/dL may be more appropriate for renal protection. On the other hand, Kanda et al. reported a U-shaped relationship between serum UA level and renal function in a prospective cohort study in Japan [16]. Furthermore, a cross-sectional study of 227,645 people in Japan also showed that hypouricemia (serum UA < 2.0 mg/dL) was associated with reduced renal function [17]. These Japanese data sets suggest that intensive reduction in serum UA may cause undesirable outcome on renal function. Obviously, it is important to determine an optimal target level of serum UA to inform clinical decision making. With this in mind, we investigated the effect of different levels of lowered UA on renal function using topiroxostat, a non-purine-type selective XOI to determine whether Intensive UA-lowering therapy (ULT) would provide better renal protection compared with standard therapy in CKD patients with hyperuricemia.

## Methods

### Design

Details of the methods of this study have previously been published [18]. This was a multicenter randomized controlled trial. The participants were randomly assigned to either the Intensive therapy group (target serum UA level ≥ 4.0 mg/dL, < 5.0 mg/dL) or the Standard therapy group (serum UA level ≥ 6.0 mg/dL, < 7.0 mg/dL). ULT was performed for 52 weeks in both groups. Registration and allocation of participants was carried out by the central registration modality using an electronic data capturing system. Participants were assigned to the groups using permutation-block randomization with the allocation factors of renin-angiotensin system (RAS) inhibitors, diabetes, and study sites. Centralized measurements were performed for all blood and urine samples.

This study was registered in the Clinical Trial Registries in Japan (UMIN000026741 and jRCTs051180146).

### Intervention

Topiroxostat was administered at a starting dose of 20 mg twice daily, with increases up to a maximum of 160 mg/day until serum UA levels reached the target range for both groups. Combined administration of benzbromarone with topiroxostat was allowed when the serum UA level did not reach the target range even at the maximum dosage of topiroxostat. Other XOIs (allopurinol and febuxostat) were prohibited during the study. Dose modification, additional administration or discontinuation of RAS inhibitors and diuretics were also prohibited during the study.

### Endpoints

The primary endpoint was change in the logarithmic value of urine albumin to the creatinine ratio (ACR) between baseline and week 52 of the treatment. Secondary endpoints were changes in serum UA, eGFR, and urinary protein, and the incidence of composite cardiovascular events and renal events. Composite cardiovascular events included newly diagnosed myocardial infarction, angina requiring revascularization, heart failure requiring hospitalization, stroke (cerebral infarction and cerebral hemorrhage), peripheral arterial disease requiring revascularization, carotid artery stenosis requiring stent placement or endarterectomy, aortic aneurysm or dissection requiring hospitalization, and sudden death. Renal events included the appearance of overt proteinuria, serum creatinine level ≥ 2.0 mg/dL with an increase of more than twofold, and renal replacement therapy (dialysis or kidney transplantation).

For the safety assessment, adverse events (AEs) including serious AEs during the study period were collected.

### Patients

The inclusion criteria in this study included fulfillment of all of the following: (1) aged ≥ 20 years, (2) eGFR ≥ 30 and < 60 mL/min/1.73 m^2^ and urinary protein < 0.5 g/gCr or ACR < 300 mg/gCr, (3) serum UA ≥ 8.0 mg/dL or ≥ 7.0 mg/dL currently under treatment with allopurinol or benzbromarone. Key exclusion criteria were as follows: (1) patients receiving febuxostat or topiroxostat, 2) history of repetitive gouty arthritis, (3) history of urolithiasis attack within 6 months, (4) obstructive uropathy, (5) active malignancy, (6) severe hepatic dysfunction, (7) diabetes with HbA1c > 8.4%, and (8) severe hypertension (SBP ≥ 180 mmHg or DBP ≥ 110 mmHg).

### Population analysis

Statistical analysis was performed with full analysis set (FAS), modified per-protocol set PPS (mPPS), and safety analysis set (SAS). The FAS was the main analysis target for effectiveness, and was defined as the population excluding any of the following participants: not meeting eligibility criteria, never received study treatment, or having no data after allocation. The PPS was defined as the population excluding participants with protocol violation from the FAS, and the Modified PPS (mPPS) was defined as the population who had achieved the target UA level at least once from week 24 to week 52 in the PPS. The SAS was defined as the population who were administered topiroxostat at least once during the study.

### Measurements

Centralized measurements of blood and urine analyses were performed by SRL Co., Ltd (Hachioji Japan:ISO15189). Xanthine oxidoreductase (XOR) activity and blood topiroxostat concentration were quantiied by Fujiyakuhin Co., Ltd. (Saitama, Japan). The concentration of neutrophil gelatinase-associated lipocalin (N-GAL) were measured using chemiluminescent microparticle immunoassays by Abbott Japan Co., Ltd. (Tokyo, Japan).

### Statistical analysis

A *p*-value of 0.05 or less (two sided) was the criterion for statistical significance. The package software SAS Ver. 9.4 was used for statistical analyses. Sensitivity analyses were performed for the mPPS in addition to FAS for the primary endpoint. For the evaluation of the primary endpoint, the amount of change in logarithmic value of urinary ACR from baseline at week 52 was compared between groups in FAS. The mixed-effects model for repeated measures (MMRM) was fitted for the analyses. For change from baseline in log ACR, in the MMRM model with baseline values as covariate, treatment group, each time point, and the interaction between each time point and treatment group as fixed effect, and participants as random effect, the upper limit of the 95% CI of the least squares mean (LSMean) of the group difference in change at 52 weeks was considered significant if it was less than zero.

To evaluate secondary endpoints, changes in the UA and eGFR (the LSMeans of the group differences in change at each time point) were calculated in the MMRM model. The baseline value was set as the covariate. The treatment group, each time point, and the interaction between each time point and the treatment group were set as fixed effects, and the participants were set as the random effect. A group difference in the categorical shifts at week 52 from baseline in proteinuria was compared with Fisher's exact test. The rates in composite cardiovascular events and renal events were estimated using the Kaplan–Meier method, and the event rates were compared between groups using log-rank tests.

## Results

### Patient characteristics

**Fig. 1:**
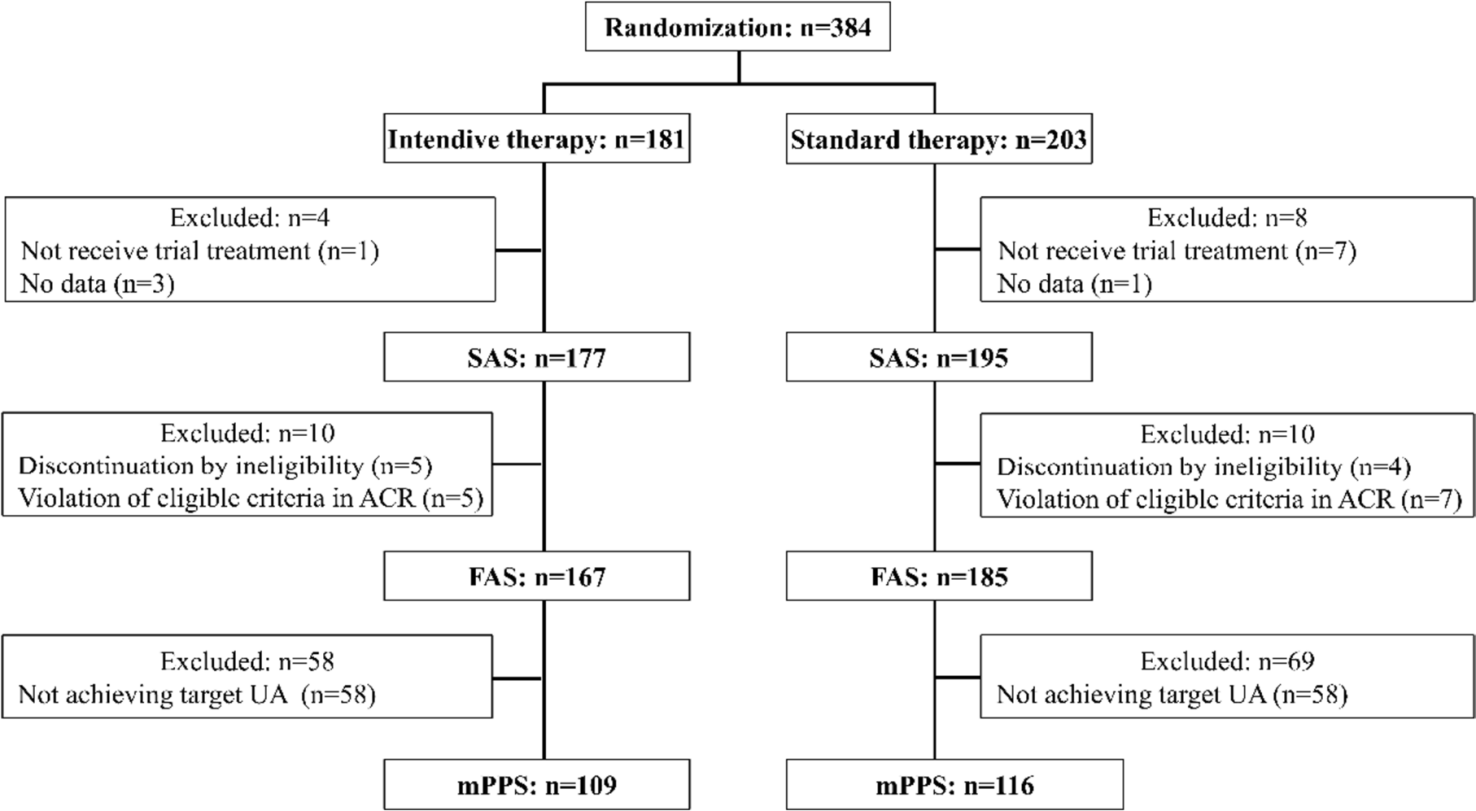
Enrollment and randomization flow. *SAS* safety analysis set, *FAS* full analysis set, m*PPS* modified per-protocol analysis set

Of the 384 patients, 181 were assigned to the Intensive therapy group and 203 to the Standard therapy group. 372 patients were included in the SAS after ineligible patients were excluded (Fig. 1). Three hundred fifty-two patients were included in the FAS after excluding ineligible patients from SAS. The mPPS that achieved the target serum UA level consisted of 225 patients, corresponding to 63.9% of the FAS.

The mean age of the FAS was 66.9 years. 81.8% of these were male (Table 1). The mean values for each measurement were 8.20 mg/dL for UA, 51.6 mL/min/1.73m^2^ for eGFR, and 60.7 mg/gCre for ACR. Hypertension and dyslipidemia were found in 73.3% and 51.4% of patients, respectively. There was no obvious difference in patient background between the Intensive therapy and the Standard therapy groups.

**Table 1:** Patient characteristics in FAS

	Total (*n* = 352)	Intensive therapy (*n* = 167)	Standard therapy (*n* = 185)
Age (year), mean ± SD	66.9 ± 13.0	66.2 ± 12.7	67.4 ± 13.1
Sex (Male), *n* (%)	288 (81.8%)	140 (83.8%)	148 (80.0%)
BMI (kg/m^2^), mean ± SD	25.0 ± 3.5	25.0 ± 3.4	25.1 ± 3.7
Uric acid (mg/dL) mean ± SD	8.20 ± 1.24	8.15 ± 1.31	8.23 ± 1.17
eGFR (mL/min/1.73m^2^), mean ± SD	51.60 ± 10.96	51.24 ± 11.31	51.93 ± 10.66
ACR (mg/gCre), mean ± SD	60.70 ± 198.36	48.23 ± 88.09	72.01 ± 260.66
Smoking, *n* (%)			
Never	137 (38.9%)	66 (39.5%)	71 (38.4%)
Past	159 (45.2%)	75 (44.9%)	84 (45.4%)
Present	56 (15.9%)	26 (15.6%)	30 (16.2%)
Alcohol, *n* (%)			
Never	82 (23.3%)	41 (24.6%)	41 (22.2%)
Past	54 (15.3%)	26 (15.6%)	28 (15.1%)
Present	216 (61.4%)	100 (59.9%)	116 (62.7%)
Comorbidity, *n* (%)			
Hypertension	258 (73.3%)	123 (73.7%)	135 (73.0%)
Diabetes	66 (18.8%)	30 (18.0%)	36 (19.5%)
Dyslipidemia	181 (51.4%)	91 (54.5%)	90 (48.6%)
Renal dysfunction			
Diabetic kidney disease	19 (5.4%)	6 (3.6%)	13 (7.0%)
Hypertensive renal sclerosis	66 (18.8%)	31 (18.6%)	35 (18.9%)
Chronic glomerulonephritis	18 (5.1%)	10 (6.0%)	8 (4.3%)
Medication, *n* (%)			
XOI	68 (19.3%)	37 (22.2%)	31 (16.8%)
Uric acid lowering drugs other than XOI	8 (2.3%)	4 (2.4%)	4 (2.2%)
Antihypertensive drugs/RAS inhibitors	244 (69.3%)	116 (69.5%)	128 (69.2%)
Diuretics	75 (21.3%)	31 (18.6%)	44 (23.8%)
Antidiabetic drugs	45 (12.8%)	20 (12.0%)	25 (13.5%)
NSAIDs	28 (8.0%)	14 (8.4%)	14 (7.6%)

*ACR* urine albumin to creatinine ratio, e*GFR* estimated glomerular filtration rate, *NSAIDs* non-steroidal anti-inflammatory drugs, *RAS* renin–angiotensin system, *XOI* xanthine oxidase inhibitor

### Primary endpoint

**Fig. 2:**
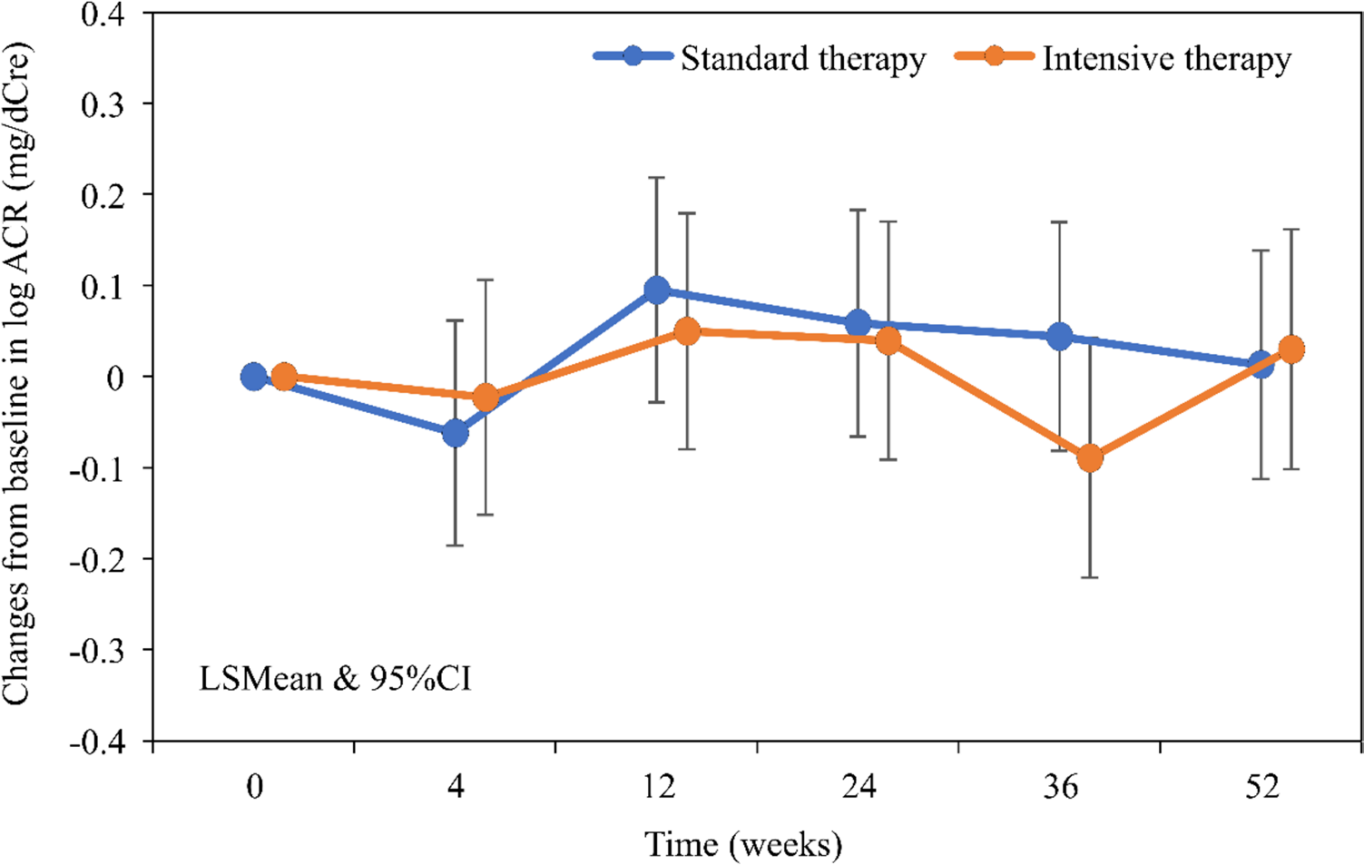
Changes from baseline in log ACR *ACR* urine albumin to creatinine ratio, *LSM*ean least square mean, 95% *CI* 95% confidence interval

Figure 2 shows changes from baseline in log ACR for both groups. There was no significant difference in change in log ACR at 52 weeks between the Intensive and the Standard therapy groups (Table 2). Supplemental Table 1 shows the results of the MMRM for the change in log ACR. The upper limit of the 95% CI for the LSMean of the group difference in log ACR change at 52 weeks was 0.196, indicating no significant difference between groups. In the same analysis for the mPPS, there was also no significant difference in change in log ACR at 52 weeks between the groups (data not shown).

**Table 2:** Changes from baseline in various measurements in FAS

	Intensive therapy	Standard therapy	Group difference
log ACR, LSMean [95% CI]			
Week 4	−0.023 [−0.152, 0.106]	−0.062 [−0.186, 0.062]	0.039 [−0.140, 0.218]
Week 12	0.05 [−0.080, 0.179]	0.095 [−0.029, 0.219]	−0.045 [−0.225, 0.134]
Week 24	0.039 [− 0.091, 0.170]	0.058 [−0.066, 0.183]	−0.019 [−0.199, 0.162]
Week 36	−0.09 [−0.221, 0.042]	0.044 [− 0.082, 0.170]	−0.134 [−0.316, 0.048]
Week 52	0.03 [−0.102, 0.162]	0.013 [−0.113, 0.139]	0.017 [−0.165, 0.199]
Uric acid, LSMean [95% CI]			
Week 4	− 1.693 [− 1.878, − 1.508]	− 1.671 [− 1.848, − 1.495]	− 0.021 [− 0.277, 0.234]
Week 12	− 2.341 [− 2.527, − 2.155]	− 2.011 [− 2.187, − 1.834]	− 0.331 [− 0.587, − 0.074]
Week 24	− 2.832 [− 3.020, − 2.645]	− 2.062 [− 2.240, − 1.884]	− 0.77 [− 1.029, − 0.512]
Week 36	− 3.01 [− 3.199, − 2.821]	− 2.124 [− 2.304, − 1.945]	− 0.886 [− 1.146, − 0.625]
Week 52	− 2.929 [− 3.118, − 2.740]	− 2.03 [− 2.210, − 1.850]	− 0.899 [− 1.160, − 0.638]
eGFR, LSMean [95% CI]			
Week 4	− 0.136 [− 1.101, 0.829]	− 0.131 [− 1.053, 0.792]	− 0.006 [− 1.341, 1.330]
Week 12	0.538 [− 0.434, 1.509]	0.808 [− 0.116, 1.731]	− 0.27 [− 1.611, 1.071]
Week 24	0.129 [− 0.850, 1.108]	− 0.017 [− 0.946, 0.912]	0.146 [− 1.204, 1.496]
Week 36	1.016 [0.031, 2.002]	0.239 [− 0.700, 1.177]	0.778 [− 0.584, 2.139]
Week 52	0.044 [− 0.943, 1.031]	− 0.304 [− 1.244, 0.637]	0.347 [− 1.016, 1.711]

*ACR* urine albumin to creatinine ratio, e*GFR* estimated glomerular filtration rate, *FAS* full analysis set, *LSMean* least mean, 95% *CI* 95% confidence interval

### Secondary endpoints

**Fig. 3:**
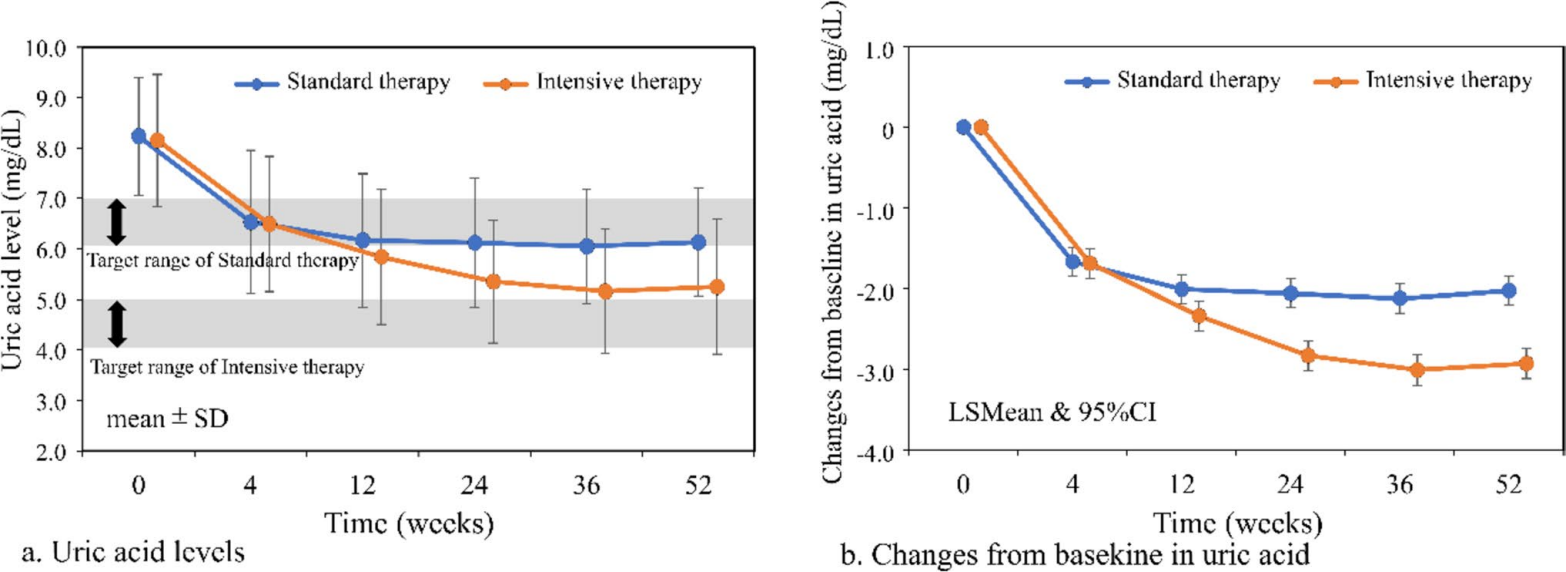
Changes in uric acid *UA* uric acid, *LSMean* least square mean, 95% *CI* 95% confidence interval, *SD*: standard deviation

**Fig. 4:**
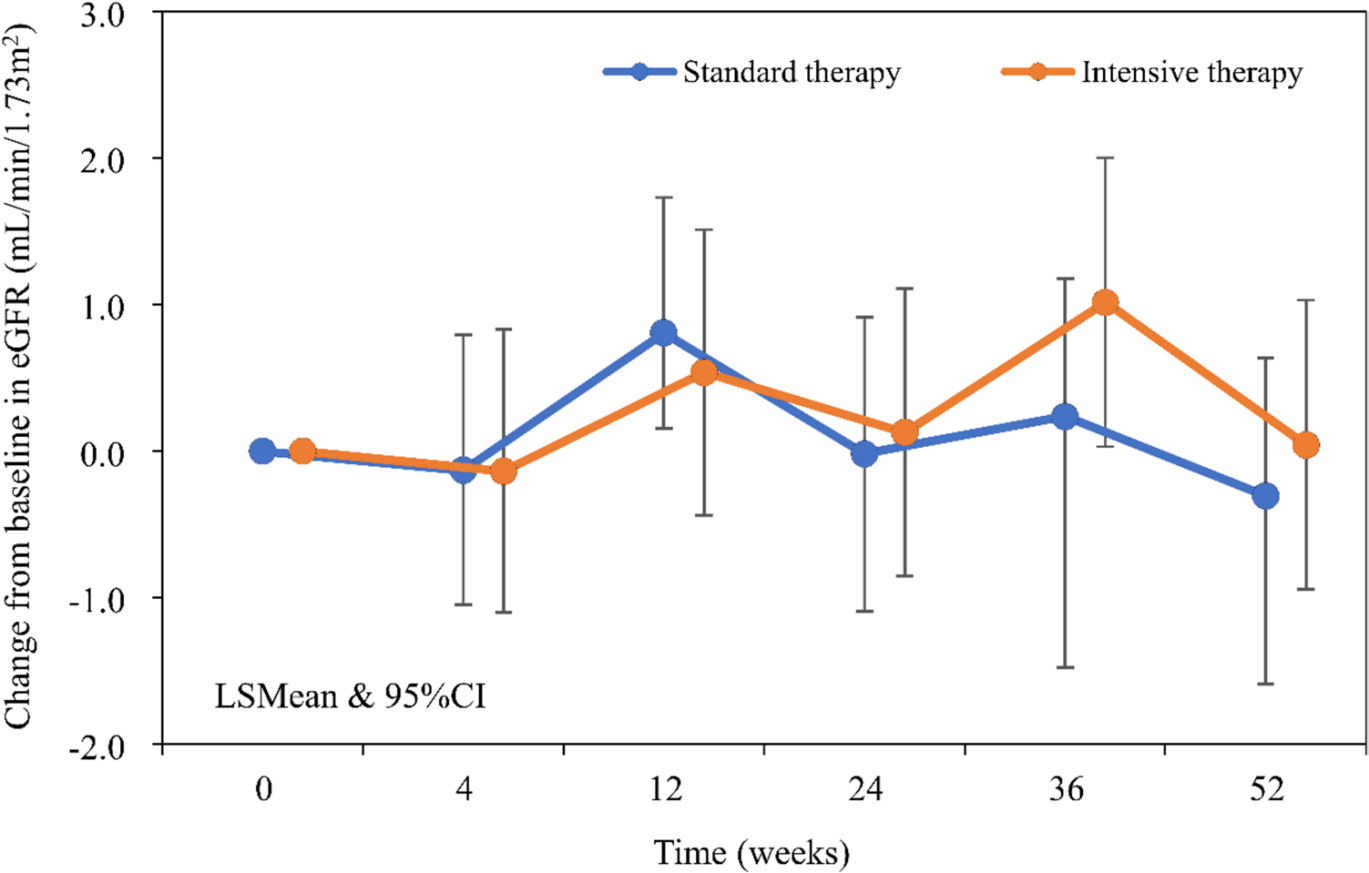
Changes from baseline in eGFR e*GFR* estimated glomerular filtration rate, *LSMean* least square mean, 95% *CI* 95% confidence interval

**Fig. 5:**
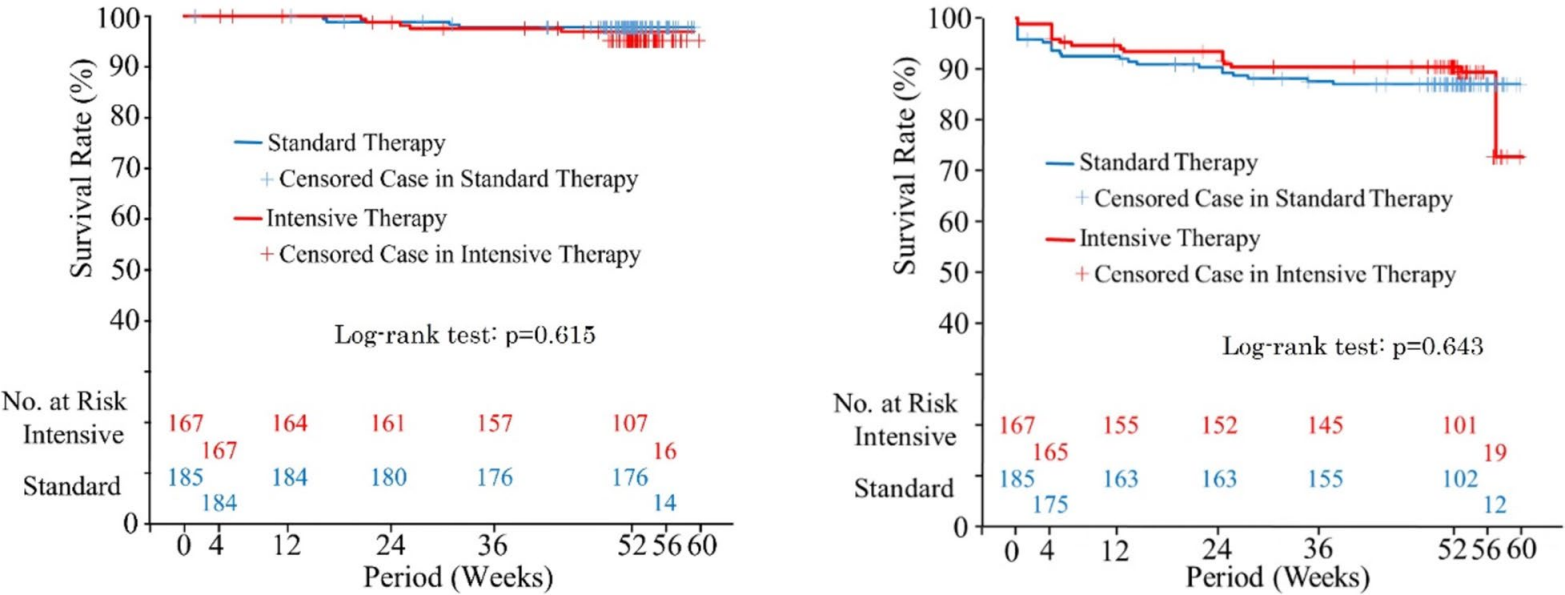
Survival analysis of cardiovascular and renal events

Figure 3a shows the change in serum UA. In the Standard therapy group, mean serum UA was 8.23 mg/dL at baseline and 6.13 mg/dL at 52 weeks with a change of − 2.10 mg/dL. In the Intensive therapy group, mean serum UA was 8.15 mg/dL at baseline and 5.25 mg/dL at 52 weeks with a change of − 2.90 mg/dL. Mean UA levels were within the target range in the Standard therapy group, but did not reach the target range in the Intensive therapy group. Changes from baseline in serum UA are shown in Fig. 3b. MMRM showed that the change from baseline in serum UA was significantly different between the two groups at 12, 24, 36, and 52 weeks (all *p* < 0.0001) (Supplemental Table 1). Changes from baseline in eGFR are shown in Fig. 4. MMRM showed that the change in eGFR from baseline was not significantly different between the two groups at any time point (Supplemental Table 1). Changes in urinary protein status were 6.0% improved, 84.7% unchanged, and 9.3% worsened in the standard therapy group, and 6.0%, 80.8%, and 13.2%, respectively, in the Intensive therapy group, with no significant difference between the two groups (Table 3). Composite cardiovascular events occurred in 2.2% (4/185) in the Standard therapy group and 3.0% (5/167) in the Intensive therapy group, with no significant difference between the two groups (*p* = 0.615) (Fig. 5a). Renal events occurred in 13.0% (24/185) in the Standard therapy group and 12.0% (20/167) in the Intensive therapy group, with no significant difference between groups (*p* = 0.6426) (Fig. 5b).

**Table 3:** Changes in urinary protein status

	Total	Improved	Unchanged	Worsened	*p*
Standard therapy, *n* (%)	183	11 (6.0%)	155 (84.7%)	17 (9.3%)	0.520
Intensive therapy, *n* (%)	167	10 (6.0%)	135 (80.8%)	22 (13.2%)

### Safety

AEs occurred in 18.3% (68/372) of patients, 22.0% (39/177) in the Intensive therapy group and 14.9% (29/195) in the standard therapy group. SAEs were found in 5.6% (10/177) in the Intensive therapy group and 6.2% (12/195) of patients in the Standard therapy group (Table 4). There was no apparent difference in SAEs between the groups.

**Table 4:** SAEs

	Total (*n* = 372)	Intensive therapy (*n* = 177)	Standard therapy (*n* = 195)
SAEs, *n* (%)	22 (5.9%)	10 (5.6%)	12 (6.2%)
Cardiac disorders			
Atrial fibrillation	1 (0.3%)	0	1 (0.5%)
Ear and labyrinth disorders			
Meniere’s disease	1 (0.3%)	1 (0.6%)	0
Gastrointestinal disorders			
Duodenal ulcer	1 (0.3%)	0	1 (0.5%)
Inguinal hernia	1 (0.3%)	0	1 (0.5%)
General disorders and administration site conditions			
Fever	1 (0.3%)	1 (0.6%)	0
Hepatobiliary disorders			
Biliary calculus	1 (0.3%)	1 (0.6%)	0
Cholecystitis	1 (0.3%)	1 (0.6%)	0
Cholelithiasis	1 (0.3%)	0	1 (0.5%)
Infections and infestations			
Pneumonia	3 (0.3%)	2 (1.1%)	1 (0.5%)
Sepsis	1 (0.3%)	1 (0.6%)	0
Urinary tract infection	1 (0.3%)	1 (0.6%)	0
Bacterial enteritis	1 (0.3%)	1 (0.6%)	0
COVID-19	1 (0.3%)	1 (0.6%)	0
Injury, poisoning and procedural complications			
Femoral neck fracture	1 (0.3%)	1 (0.6%)	0
Fracture	1 (0.3%)	0	1 (0.5%)
Joint dislocation	1 (0.3%)	1 (0.6%)	0
Metabolism and nutrition disorders			
Dehydration	1 (0.3%)	1 (0.6%)	0
Hyperglycemia	1 (0.3%)	0	1 (0.5%)
Musculoskeletal and connective tissue disorders			
Foot deformity	1 (0.3%)	1 (0.6%)	0
Neoplasms benign, malignant and unspecified			
Pancreatic cancer	1 (0.3%)	0	1 (0.5%)
Rectal cancer	1 (0.3%)	0	1 (0.5%)
Rectal sigmoid cancer	1 (0.3%)	0	1 (0.5%)
Nervous system disorders			
Syncope	1 (0.3%)	0	1 (0.5%)
Respiratory, thoracic and mediastinal disorders			
Respiratory failure	1 (0.3%)	0	1 (0.5%)

*SAEs* serious adverse events

## Discussion

The significance of microalbuminuria in this study is its position as a marker for vascular damage, and the purpose of this study is to examine whether vascular endothelial damage can be improved by strongly lowering uric acid. Although there is a possibility that mild vascular endothelial damage may be improved by lowering serum uric acid levels, it is difficult to expect a protective effect against moderate or more severe damage. Therefore, when selecting patients, we deliberately excluded patients with overt albuminuria and selected patients with minimal or almost zero albuminuria. In this study, there were no significant differences in changes in renal function, cardiovascular events, or renal events between the Intensive therapy and the standard therapy groups. However, several previous studies have shown significant results, although some studies did not. Regarding the reports from randomized controlled trial of XOI, Hosoya et al. reported that topiroxostat significantly reduced ACR compared to placebo in patients with stage 3 CKD [9]. Stack et al. also reported that the combination therapy of febuxostat and vernurad, a urate transporter inhibitor, significantly reduced ACR compared to the placebo group [19]. With respect to the report by Hosoya et al., serum UA decreased from 8.47 mg/dL at baseline to 4.62 mg/dL in the topiroxostat group and did not change from 8.47 mg/dL at baseline in the placebo group, with a mean UA difference of 3.85 mg/dL between the two groups at final measurement [9]. Similarly, Stack et al. reported a difference in mean serum UA of 3.30 mg/dL between the two groups. In our study, the change in mean UA was from 8.15 mg/dL to 5.25 mg/dL in the Intensive therapy group and from 8.23 mg/dL to 6.13 mg/dL in the Standard therapy group, with a mean difference of 0.88 mg/dL between the two groups at the final measurement, and this difference of UA between groups was relatively small compared to the aforementioned studies. This is presumably one of the reasons for the lack of difference in the change of log ACR between the groups in our study.

The target UA range for the Intensive therapy group in our study was 4.0 to 5.0 mg/dL, but the actual mean UA at the final measurement was 5.25 mg/dL in the FAS. The reason for this is that the recommended range of UA for CKD patients in Japanese guidelines is 6.0 mg/dL or less [13], and it is possible that the physicians in this study hesitated to lower the UA levels to 5.0 mg/dL or less, which is lower than the treatment guideline. Badve et al. reported that 104 weeks of urate-lowering therapy with allopurinol resulted in an average reduction of 2.7 mg/dl versus placebo, but did not reduce albuminuria or slow the decline in eGFR compared with placebo [20]. In our study, same as Badve's Contrary to expectations, lowering uric acid levels did not improve microalbuminuria. Furthermore, in our study, a before-and-after comparison of uric acid levels showed a decrease of − 2.929 mg/d in the intensive group, but no improvement in microalbuminuria or eGFR was observed. A possible reason is the effect of RAS inhibitors. RAS inhibitors were administered to approximately 69% of the cases, and it is likely that RAS inhibitors had a large effect on protecting the vascular endothelium. Under an environment where RAS inhibitors are sufficiently effective, it would have been difficult to obtain effects that exceed those of RAS inhibitors.

During the course of this study, Blood pressure did not show significant changes in both groups. The average blood pressure in intensive and standard group were 131.1 mmHg and 131.9 mmHg at baseline, 129.7 mmHg and 130.4 mmHg at week 52, respectively. We compared the rate of XOR activity reaching below the detection limit at 52 weeks. The rate was significantly higher in the active therapy group, at 59.2% in the active therapy group and 47.4% in the standard therapy group (*p* = 0.041).

The incidence of composite cardiovascular events at 1-year follow-up was 3.0% (5/167) in the Intensive therapy group and 2.2% (4/185) in the standard therapy group, with no significant difference between the groups. One reason for the lack of difference in the incidence rates between the groups may be that the follow-up period was one year, and the incidence rates were relatively low in both groups.

## Limitations

This study has several limitations. The mean serum UA in the Intensive therapy group did not reach the target range. Follow-up period in this study was 1 year, and the incidence of various events was low, which may have made it difficult to detect differences between the groups. The results of this study should be interpreted with these considerations in mind.

## Conclusion

In CKD patients with hyperuricemia, there was a significant difference in serum UA levels between the Intensive therapy group and the Standard therapy group. However, there was no significant difference in log ACR change between the groups. This study did not reveal the benefit of Intensive ULT to improve albuminuria levels.

### Supplementary Information

Below is the link to the electronic supplementary material.

Supplementary file1 (DOCX 16 KB)
