# Supplementary material for: Multicenter randomized controlled trial of intensive uric acid lowering therapy for CKD patients with hyperuricemia: TARGET-UA
Source: Clin Exp Nephrol. 2024 Mar 26;28(8):764–72. doi: 10.1007/s10157-024-02483-w (PMC11266370; doi:10.1007/s10157-024-02483-w)
Supplement: Supplementary file 1 — Supplementary file1 (DOCX 16 KB) [file 10157_2024_2483_MOESM1_ESM.docx]

Supplementary Table 1. MMRM analysis of changes from baseline in each variable in FAS

| Variable | | Parameter estimated [95% CI] | p-value |
| --- | --- | --- | --- |
| log ACR | |  |  |
|  | Intercept | 0.257 [0.080, 0.434] | 0.005 |
|  | log ACR at Baseline | -0.116 [-0.162, -0.070] | <0.001 |
|  | Intensive Therapy | 0.039 [-0.140, 0.218] | 0.668 |
|  | Week12 | 0.157 [0.045, 0.269] | 0.006 |
|  | Week24 | 0.120 [-0.006, 0.247] | 0.063 |
|  | Week36 | 0.106 [-0.021, 0.233] | 0.101 |
|  | Week52 | 0.075 [-0.046, 0.196] | 0.225 |
|  | Intensive Therapy * Week12 | -0.084 [-0.247, 0.078] | 0.306 |
|  | Intensive Therapy * Week24 | -0.058 [-0.242, 0.125] | 0.534 |
|  | Intensive Therapy * Week36 | -0.173 [-0.356, 0.010] | 0.064 |
|  | Intensive Therapy * Week52 | -0.022 [-0.197, 0.153] | 0.805 |
| Uric acid | |  |  |
|  | Intercept | 4.009 [3.350, 4.668] | <0.001 |
|  | UA at Baseline | -0.694 [-0.771, -0.616] | <0.001 |
|  | Intensive Therapy | -0.021 [-0.277, 0.234] | 0.87 |
|  | Week12 | -0.339 [-0.508, -0.171] | <0.001 |
|  | Week24 | -0.391 [-0.594, -0.187] | <0.001 |
|  | Week36 | -0.453 [-0.644, -0.263] | <0.001 |
|  | Week52 | -0.359 [-0.549, -0.169] | <0.001 |
|  | Intensive Therapy * Week12 | -0.309 [-0.554, -0.065] | 0.013 |
|  | Intensive Therapy * Week24 | -0.749 [-1.044, -0.454] | <0.001 |
|  | Intensive Therapy * Week36 | -0.864 [-1.140, -0.589] | <0.001 |
|  | Intensive Therapy * Week52 | -0.877 [-1.153, -0.602] | <0.001 |
| eGFR | |  |  |
|  | Intercept | 3.810 [1.280, 6.339] | 0.003 |
|  | eGFR at Baseline | -0.076 [-0.122, -0.031] | 0.001 |
|  | Intensive Therapy | -0.006 [-1.341, 1.330] | 0.993 |
|  | Week12 | 0.938 [-0.044, 1.921] | 0.061 |
|  | Week24 | 0.114 [-0.928, 1.155] | 0.831 |
|  | Week36 | 0.369 [-0.654, 1.392] | 0.479 |
|  | Week52 | -0.173 [-1.143, 0.797] | 0.727 |
|  | Intensive Therapy * Week12 | -0.265 [-1.688, 1.159] | 0.715 |
|  | Intensive Therapy * Week24 | 0.152 [-1.358, 1.661] | 0.844 |
|  | Intensive Therapy * Week36 | 0.783 [-0.697, 2.264] | 0.299 |
|  | Intensive Therapy * Week52 | 0.353 [-1.051, 1.757] | 0.622 |

ACR: urine albumin to creatinine ratio, eGFR: estimated glomerular filtration rate, MMRM: mixed-effects model for repeated measures, Intensive Therapy * Week X: Interaction between Intensive therapy and measurement week
